# Phospholipids: Key Players in Apoptosis and Immune Regulation

**DOI:** 10.3390/molecules14124892

**Published:** 2009-11-30

**Authors:** Ricardo A. Chaurio, Christina Janko, Luis E. Muñoz, Benjamin Frey, Martin Herrmann, Udo S. Gaipl

**Affiliations:** 1 Department for Internal Medicine 3, University Hospital Erlangen, Friedrich-Alexander University of Erlangen-Nuremberg, 91054 Erlangen, Germany; 2 Department of Radiation Oncology, University Hospital Erlangen, Friedrich-Alexander University of Erlangen-Nuremberg, 91054 Erlangen, Germany

**Keywords:** phosphatidylserine, phospholipids, apoptosis, clearance, phagocytosis, immune modulation

## Abstract

Phosphatidylserine (PS), a phospholipid predominantly found in the inner leaflet of eukaryotic cellular membranes, plays important roles in many biological processes. During apoptosis, the asymmetric distribution of phospholipids of the plasma membrane gets lost and PS is translocated to the outer leaflet of the plasma membrane. There, PS acts as one major “eat me” signal that ensures efficient recognition and uptake of apoptotic cells by phagocytes. PS recognition of activated phagocytes induces the secretion of anti-inflammatory cytokines like interleukin-10 and transforming grow factor-beta. Deficiencies in the clearance of apoptotic cells result in the occurrence of secondarily necrotic cells. The latter have lost the membrane integrity and release immune activating danger signals, which may induce inflammatory responses. Accumulation of dead cells containing nuclear autoantigens in sites of immune selection may provide survival signals for autoreactive B-cells. The production of antibodies against nuclear structures determines the initiation of chronic autoimmunity in systemic lupus erythematosus. Since PS on apoptotic cells is an important modulator of the immune response, natural occurring ligands for PS like annexinA5 have profound effects on immune responses against dead and dying cells, including tumour cells. In this review we will focus on the role of PS exposure in the clearance process of dead cells and its implications in clinical situations where apoptosis plays a relevant role, like in cancer, chronic autoimmunity, and infections. Relevance of other phospholipids during the apoptosis process is also discussed.

## 1. Introduction

The plasma membrane in eukaryotic cells is characterized by an asymmetrical distribution of phospholipids, the most abundant lipid components in membranes. Aminophospholipids, like phosphatidylserine (PS) and phosphatidylethanolamine (PE), are generally enriched in the cytoplasmic leaflet, while phosphatidylcholine (PC), sphingomyelin (SM), and glycosphingolipids are mainly located on the exoplasmic leaflet. Additionally minor phospholipids, such as phosphatidic acid and phosphatidylinositol (PI), are also found on the cytoplasmic face. Such membrane asymmetry is widely observed in eukaryotes from yeast to mammalian cells [1,2]. Although PS is quantitatively a minor component, it is widely distributed in cellular organelles, suggesting an essential structural role in biological membranes [3]. In quiescent cells, PS is mainly located on the cytoplasmic side of the plasma membrane. Once cells get activated, PS is transiently and rapidly externalized on the cell surface as occurs on activated platelets during coagulation and platelet aggregation [4,5]. PS can be also present at the surface of exosomes derived from platelets and dendritic cells (DCs) [6], on viable monocytes [7], on the surface of mature macrophages [8], on nuclei expelled from erythroid precursor cells [9]; on activated B cells [10], in the nuclear matrix [11,12], and as soluble PS (sPS) derived from cancer cells [13]. Under certain pathological and physiological situations (e.g., in cancer), PS can be also spontaneously exposed as occurs in the vascular endothelial cells of vasculature in tumours [14,15,16,17].

The normal asymmetrical architecture of the membrane can be perturbed permanently when cells undergo programmed cell death (apoptosis), a coordinated process of cell suicide that comprises the controlled elimination of activated, damage, or senescent cells [18]. During apoptosis, PS is exposed on the outer leaflet of the membrane [19,20] demonstrated by its’ biochemical detection on the surface of apoptotic but not non-apoptotic lymphocytes [21]. The evolutionary origin of PS exposure during cell death has been estimated over 600 million years ago including a wide range of organisms from nematodes to mammalians [22]. In this review we will focus on the importance of PS exposure in the clearance process of dying cells and its implications in clinical situations where apoptosis plays a relevant role, like cancer, chronic autoimmunity, and infections. The relevance of other phospholipids during apoptosis and corpses removal process is also discussed.

## 2. Phospholipids in Biological Membranes

**Figure 1:**
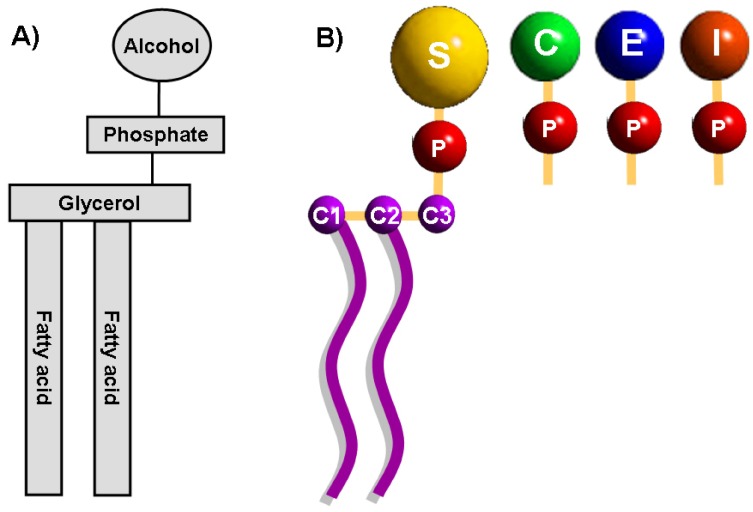
General structure of membrane glycerophospholipids.(A) The structure of glycerophospholipids consists of a glycerol backbone, two fatty acid chains, and a phosphorylated alcohol. (B) The C-3 hydroxyl group of the glycerol backbone is sterified to phosphoric acid which can also be sterified to the hydroxyl group of one of several alcohols moieties like choline, ethanolamine, inositol and serine, giving rise to the most prominent phospholipids of cellular membranes: phosphatidylcholine, phosphatidylethanolamine, phosphatidylinositol and phosphadylserine. C: choline, E: ethanolamine, I: inositol, P: phosphate group, S: serine.

Phospholipids are abundant in all biological membranes and derive from either glycerol, a three-carbon alcohol or sphingosine, a long-chain unsaturated amino alcohol [23]. Phospholipids derived from glycerol are called phosphoglycerides which consist of a glycerol backbone, two fatty acid chains, and a phosphorylated alcohol (Figure 1). The hydrocarbon chain of fatty acids is un-branched and the spatial conformation of double bonds in unsaturated fatty acids is almost always cis. The length and the degree of unsaturation of fatty acids in the membrane have an important effect on the fluidity. 

In phosphoglycerides, the hydroxyl groups at C-1 and C-2 of glycerol are sterified with carboxyl groups of fatty acid chains. The C-3 hydroxyl group of the glycerol backbone is sterified to phosphoric acid. The resulting compound, the phosphatidic acid (PA) or phosphatidate, is a vital lipid present in the cell at very low levels which functions as a biosynthetic precursor for the formation (directly or indirectly) of all acylglycerol lipids required by the cell [24,25]. The phosphate group of the PA can also be sterified to the hydroxyl group of one of several alcohols moieties like choline, ethanolamine, glycerol, inositol and serine, producing the principal known phospholipids in the cell. Mammalian cell membranes contain more than 1000 different phospholipids as the result of distinct fatty acyl chains esterified with C-1 and C-2 positions of the glycerol backbone. The distribution, quantity and functionality in the cell are complex (see [26] for an extensive review). Phosphatidylcholine, is abundant in mammalian cell membranes, constituting about 40-50% of total phospholipids while phosphatidylethanolamine (PE) comprises 20-50%. PS is quantitatively a minor membrane anionic phospholipid representing up to 10% of total phospholipids [27].

Other important phospholipids include sphingomyelin, phosphatidylinositol, and the mitochondria-specific phospholipid cardiolipin (CL), also known as diphosphatidylglycerol. Cardiolipin is mainly located and synthesized on the mitochondrial inner membrane [28,29].

## 3. Major Membrane Changes during Early Apoptosis

Apoptosis is a coordinated physiological process of programmed cell death encompassing a series of biochemical events that result in the death and elimination of the cell. Apoptosis is vital for embryologic development and maintenance of tissue homeostasis in multicellular organisms and is characterised by specific morphological changes of the dying cells; namely, loss of membrane asymmetry, cytoskeleton remodelling, plasma membrane blebbing, loss of the mitochondrial membrane potential, caspase activation, chromatin condensation, and DNA fragmentation. Phospholipid translocation in cellular membranes during apoptosis has been considered one of the most important markers of the initial phases of apoptosis [30]. Furthermore, PS exposure on the cell surface and CL movement among the mitochondrial membranes are considered as key events involved in the programmed cell death process and are summarised as follows.

### 3.1. PS translocation in apoptosis

PS regulation and exposure after the initiation of apoptosis has been associated at least with three possible mechanisms: (1) P-type ATPases (flippases or translocases) are a large family of transmembrane proteins responsible for the active transport of aminophospholipids analogues from the outer leaflet to the inner leaflet of the plasma membrane [31,32]. Inside this group, the type IV P-type ATPases (P4 ATPases) have been shown to be major transporters of aminoglycerophospholipids that are important for the maintenance of the asymmetric distribution in biological membranes. Currently 14 genes have been identified encoding P4-ATPases, one of them being the ATP8B1. The latter is involved in translocation of PS [33,34,35]. Inactivation of these translocases during apoptosis results in ‘randomization’ of the membrane leaflets and exposure of PS on the outer leaflet due to an inadequate re-transport of PS to the inner leaflet. (2) Scramblases are type II membrane proteins with a conserved calcium-binding C-terminal domain, involved in the loss of the membrane asymmetry during cellular processes involving an increase in the cytoplasmic calcium levels (e.g. cell activation, injury, blood coagulation and apoptosis). Scramblases are ATP-independent translocators of phospholipids localized in the membrane, involved in the rapid flip from either side of the bilayer to the other. Activated scramblases facilities a rapid bidirectional movement of lipids, regardless of headgroup, and have been suggested to move PS bi-directionally across the membrane [36]. However, the scramblase(s) involved in PS exposure during apoptosis as well as the mechanisms involved in this process need further investigations. (3) ATP Binding Cassette (ABC)-transporters consists of a large family of ATP driven channels, which transport a variety of substrates (e.g. amino acids, inorganic ions, peptides, drugs, lipids, metals, proteins, metabolic products, saccharides, sterols) into and out of the cell. ABC-transporters can also be involved in intracellular compartmental transport [37,38,39]. Studies in *C. elegans* identified the ced-7 gene which encodes a protein belonging to the ABC-transporters family. Its mammalian homolog, ABCA1, was tested to contribute to the outward translocation of PS on apoptotic cell surface. Experiments in ABC1-null mice showed a reduced externalization of PS in response to Ca^2+^ stress, defective engulfment of apoptotic cells by ABC1-deficient macrophages, and lower PS exposure in ABC1-deficient apoptotic thymocytes in comparison to wild type conditions [40]. 

We conclude that PS translocation is mainly achieved by a concerted action of P-type ATPases, scramblases, and (ABC)-transporters. Scramblases are the less specific transporters facilitating a bidirectional translocation of lipids regardless of its headgroup. P-type ATPases translocate PS to the inner leaflet of the membrane while ABC1-transporter moves PS to the surface of the cell in response to calcium signals even though ATP biosynthesis starts to decrease. 

### 3.2. Cardiolipin translocation in apoptosis

**Figure 2:**
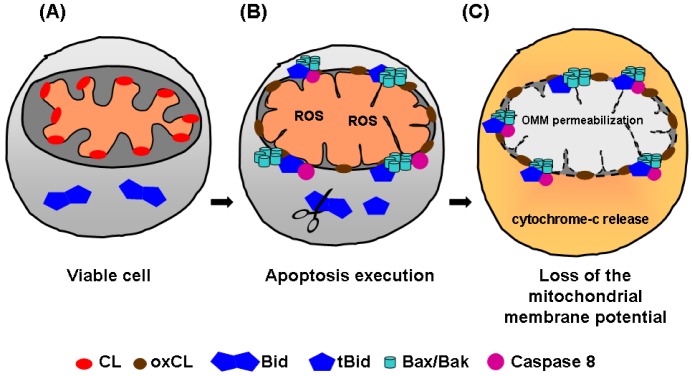
Translocation of cardiolipin among mitochondrion membranes during apoptosis.(A) In viable cells, CL is normally located in the IMM. (B) During apoptosis execution, CL is oxidated and translocated from the IMM to the OMM, acting as a central signal integrator for multiple proteins (e.g. tBid, cytochrome c, and caspase 8) and providing recognition and binding sites for Bcl-2 pro-apoptotic proteins (e.g. Bax/Bak). (C) Finally, this leads to OMM permeabilization and cytochrome c release into the cytosol. CL: cardiolipin, oxCL: oxidated cardiolipin, OMM: outer mitochondrial membrane, ROS: reactive oxygen species.

Mitochondria are complex organelles consisting of two membranes: the outer mitochondrial membrane (OMM) and the inner mitochondrial membrane (IMM). The IMM is organized in two morphologically distinct domains: the inner boundary membrane (IBM), closely opposed to the OMM, and the *cristae* membrane (CM). The CMs are invaginations of the IBM into the matrix space [41,42,43,44]. Mitochondria possess a dynamic structure, which can change depending of factors including the cellular bio-energetic state, cell cycle, and apoptosis. CL is a mitochondria-specific phospholipid predominantly located in the inner leaflet of the IMM [45], and particularly at contact sites formed between the inner and outer mitochondria membranes, where CL (~25%) and PE (~25%) levels are increased [46,47,48,49,50]. CL has also been identified in the OMM, but in low levels (~4%) [51,52]. During apoptosis, an extensive remodeling of the mitochondrial membranes occurs (Figure 2) [53], CL is translocated from the IMM to the OMM, providing recognition and binding sites for the Bcl-2 family proteins, like Bid, tBid, and Bax. Consequently, cytochrome c release is induced from the OMM to the cytosol. CL intra-mitochondrial movements occur very early during apoptosis, even before changes in mitochondrial membrane potential and PS exposure [54]. Membrane redistribution of CL is a process involving flip-flop across the IMM and the OMM, as well as an interbilayer transfer from the IMM to the OMM. These mechanisms require the participation of various processes like the formation of contact sites (local fusion areas of the IMM and OMM) [55]. Furthermore, lipid-transfer-activity molecules with capacity to induce lipid movement across bilayers are involved. Examples for those molecules are Bid and tBid) [56,57,58,59], the pro-apoptotic protein Bax [60], the mitochondrial creatine kinase (MtCK), as well as the nucleoside diphosphate kinase (NDPK-D) [61,62]. Protein transporters, specifically the phospholipid scramblase 3 (PLSCR3), play also an important role in the translocation of CL among mitochondrial membranes [63,64].

During the execution of programmed cell death, CL acts as a central signal integrator for multiple proteins, where interactions with tBid, cytochrome c, and caspase 8 play a crucial role (see [55] for an extensive review). Specifically, CL provides specificity for targeting of tBid interaction to the mitochondria [65], is necessary for the Bax/Bak oligomerization [66], *cristae* remodelling, and OMM permeabilization inducing cytochrome c release [67]. Importantly, CL oxidation is an essential step in the release of cytochrome c, during apoptosis [68]. It has been proposed that CL provides an activation platform for the pro-caspase-8 processing and production of the active form of caspase 8 [55]. Interestingly, activation of the Fas receptor induced translocation of active caspase-8 to the mitochondria [69]. CL is also present in mitochondrial raft-like microdomains, which could represent preferential sites where some key reactions can be catalyzed, contributing to cell death execution steps. It has been hypothesized that the role for CL in “raft-like” microdomains could be to anchor caspase-8 at contact sites between inner and outer membranes, facilitating its self-activation, Bid cleavage and apoptosis execution [70]. In overall, CL binding to cytochrome c, Bid, tBid, and caspase-8 are shown to be essential interactions for apoptosis progression.

From all above, we conclude that the asymmetry loss during the early phases of apoptosis, due to PS and CL movements, has important and different consequences. CL redistribution takes places first, resulting in targeting of tBid to the mitochondria, Bax/Bak oligomerization, OMM permeabilization, cytochrome c release, and finally to the loss of the mitochondrial membrane potential. The former processes diminish and affect cellular processes dependable of ATP-energy consumption (i.e., flippases). Afterwards, PS is translocated to the external cell membrane, acting as a main “eat me” signal for phagocytes, “switching on” the clearance process. 

## 4. How Does Apoptotic Cell Clearance Occur?

Clearance of apoptotic cells is a complex and multi-step process that comprises at least the following steps:

(1) *Attraction:* finding of apoptotic cells by phagocytes through the recognition of apoptotic cell-derived chemo-attractants (“find me” signals).
(2) *Recognition and engulfment:* identification of the abnormal membrane changes like PS exposure with higher lateral mobility, modifications in the glycosilation pattern of the glycocalix, and/or binding of specific bridging molecules (“eat me” signals). A positive recognition leads to phagocytosis, inspection (checking for pathogens), and degradation of the engulfed material.
(3) *Immune down regulation:* production of anti-inflammatory cytokines (“tolerate me” signals like IL-10 and TGF-ß).

### 4.1. Attraction of the phagocyte: Phospholipids as “find me” signals

Phagocytes have to find apoptotic cells on time before its membrane disrupts leading to the release of dangerous internal substances generating an undesirable immflamatory response. Phagocytes are normally not located in the immediate neighborhood of apoptotic cells so that secretion of chemotactic factors attracting monocytes and macrophages is very likely. Indeed, several attracting factors involved in the recruitment of phagocytes towards dying cells have been reported. We will focus in this review on phospholipids acting as attractants. Lysophosphatidylcholine (LPC) and sphingosine-1-phosphate (S1P) have been considered as mediators during apoptotic cell clearance. LPC is released from apoptotic cells by the caspase-3 mediated activation of the calcium- independent phospholipase A2 (iPLA_2_), and stimulates the attraction of monocytic cells and macrophages [71]. Using RNA interference and expression studies, it was demonstrated that the G-protein-coupled receptor G2A is involved in the chemotaxis of monocytic cells, suggesting LPC and G2A as an important receptor/ligand system for the attraction of phagocytes to apoptotic cells [72]. LPC has been also involved in the apoptotic recognition, acting as “eat me” signal as well, leading to the hypothesis of a bivalent function for LPC in the clearance process [73].

Sphingosine-1-phosphate (S1P) is a bioactive lipid involved in the regulation of important cellular processes, including cytoskeleton rearrangements, growth, motility, and survival [74]. S1P is released from apoptotic cells inducing chemotaxis of monocytic THP-1 and U937 cells, as well as primary monocytes and macrophages [75]. Interestingly, apoptotic Jurkat and U937 cells may upregulate sphingosine kinase 1 (SphK1) to produce and secrete S1P attracting scavenger cells to engulf them. There are many open questions regarding the biology of “find me” signals that remain to be clarified: Are other phospholipids or its derivatives involved in the recruitment of phagocytes, which receptors and signaling pathways are implicated, and can “find me” signals be targeted for therapeutic purposes?

### 4.2. Recognition of apoptotic cells: PS as “eat me” signal

**Figure 3:**
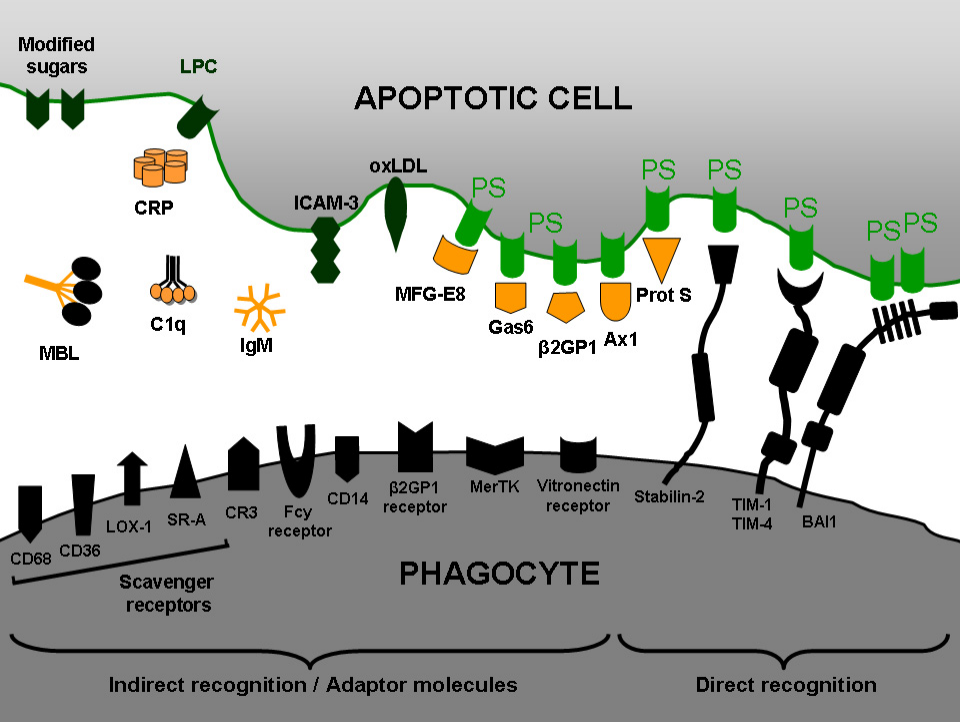
Receptors and adaptor molecules involved in apoptotic cell recognition and engulfment.Phosphatidylserine (PS) exposure enables the recognition of apoptotic cells by phagocytes. PS can be directly bound by either specific PS-receptors, such as brain-specific angiogenesis inhibitor 1 (BAI1), stabilin-2, and the T-cell immunoglobulin mucin (TIM) proteins (TIM1 and TIM4), or indirectly via bridging molecules like annexin A1 (anx A1), β2-glycoprotein I (β2GPI), the growth arrest-specific 6 (gas6), the milk-fat globule EGF-factor 8 (MFG-E8), and protein S (prot S). Other molecules also involved in the recognition of apoptotic cells are: complement protein C1q, c-reactive protein (CRP), Immunoglobulin M (IgM), mannose binding lectin (MBL), Intercellular Adhesion Molecule 3 (ICAM-3), oxidized low-density lipoprotein particle (OxLDL), lysophosphatidyl choline (LPC), lipopolysaccharide receptor (CD14), vitronectin receptor, complement receptor 3 (CR3), Mer tyrosine kinase (MerTK), Fcγ-receptor, and β2GPI receptor. Scavenger receptors involved in apoptotic cell recognition are: the lectin-like oxidized low-density lipoprotein particle receptor 1 (LOX-1), CD36, CD68, and the class A macrophage scavenger receptor (SR-A).

The recognition of PS on the outer leaflet of the plasma membrane represents the key signal for triggering phagocytosis of both, apoptotic as well as necrotic cells [76,77,78]. PS recognition occurs in a stereo specific manner (L-, but not D-phosphoserine) [79]. However, it is clear that PS is recognized by either receptors directly as a “nude” lipid, or in combination with other soluble proteins working as “bridge” or “adaptor molecules” between the phagocytes and PS on target cells. Several adaptor molecules involved in this process have been studied so far, including milk fat globule protein MFG-E8 [80], growth arrest specific gene product GAS-6 (ligand for the receptor tyrosine kinase MerTK) [81], β-2-glycoprotein-1 [82], C-reactive protein [83], serum-derived protein S [84], and annexin I [85]. These and other adaptor molecules mediate recognition and uptake of dying cells by macrophages acting as intermediaries (Figure 3). Several macrophage scavenger receptors may also interact either directly with PS or through adaptor molecules on the surface of the apoptotic cells [86]. The search for PS receptors represents one of the main challenges for many researchers worldwide. Fadok and coworkers identified a surface protein on macrophages and assumed that it represented a receptor for PS (PSR) [87]. However, this putative PSR has been ruled out as a surface receptor [88,89]. Other authors using retrovirus-mediated expression cloning system and a cDNA library from mouse peritoneal macrophages have identified Tim4 (T-cell immunoglobulin and mucin-domain-containing molecule) as one important PSR [90]. Tim4 is a type I transmembrane protein that binds apoptotic cells through PS recognition via its immunoglobulin domain. Additionally, expression of Tim4 in fibroblasts enhanced their ability to engulf apoptotic cells**.** Interestingly, among other Tim family members only Tim1, but neither Tim2 nor Tim3, was found to bind PS. Supporting these data, it has been demonstrated that TIM-4 and TIM-1 specifically bind PS on the surface of apoptotic cells [91]. Moreover, TIM-4 was found to be expressed on human and mouse macrophages as well as dendritic cells. Crystal structure analysis of murine TIM-4 identified a metal-ion-dependent ligand binding site in the immunoglobulin (Ig) domain as the PS-binding site for the TIM4 receptor [92].

Stabilin-2 has been identified as a multifunctional scavenger receptor involved in the endocytosis of modified LDL and glycation end products [93,94]. It contains a large extracellular domain that consists of seven FAS1 domains, one X-link domain, and four epidermal growth factor (EGF)-like domain repeats (EGFrp). Stabilin-2 expression has been reported in human and mouse spleen, human monocyte-derived macrophages, alveolar macrophages, and several macrophage cell lines [95]. This receptor recognizes also aged and apoptotic cells mediating its engulfment. The down-regulation of stabilin-2 expression in macrophages significantly inhibited phagocytosis of apoptotic cells. Employing the agonistic antibody anti-stabilin-2 instead of the natural ligand PS, comparable anti-inflammatory responses were obtained. Interestingly, over-expression of stabilin-2 in fibroblasts significantly enhanced both binding and engulfment of aged, but not normal, red blood cells (RBC) [96]. Moreover, EGFrp in stabilin-2 can directly and specifically recognize PS and competitively inhibit the uptake of aged RBCs and apoptotic cells via direct and preferential interaction with PS [97].

Another important receptor involved in the recognition of PS is the brain-specific angiogenesis inhibitor 1 (BAI1). BAI1 is a receptor upstream of the ELMO/Dock180/Rac signaling module that has the ability to bind PS on apoptotic cells [98]. In one hand, ELMO and Dock180 work together as a guanine nucleotide exchange factor (GEF) for the small GTPase Rac, regulating the phagocyte actin cytoskeleton during engulfment of apoptotic cells [99,100,101]. In the other hand, BAI1 is a seven-transmembrane protein that belongs to the adhesion-type G-protein-coupled receptor family, with an extended extracellular region [102,103]. The receptor BAI1 functions as engulfment receptor for both recognition and subsequent internalization of apoptotic cells. The thrombospondin type 1 repeats within the extracellular region of BAI1 are involved in the direct binding to PS. BAI1 forms a trimeric complex with ELMO and Dock180, and functional studies have suggested that BAI1 cooperates with ELMO/Dock180/Rac pathway to promote maximal engulfment of apoptotic cells. Moreover, expression of BAI1 in fibroblasts enhanced both binding and engulfment of apoptotic thymocytes [98]. 

A plethora of molecules are associated to PS recognition and binding on the surface of apoptotic cells. However, many questions remain unknown. How does PS interact with so many receptors on various cells, is PS recognized by each one of these diverse molecular structures as a monomer or like multimer complexes in the plasma membrane, are there further receptors to be described, and which signaling pathways are involved after PS recognition?

### 4.3. Immune down regulation after uptake of apoptotic cells

A hallmark of the clearance of apoptotic cells is the non-inflammatory and normally non-immunogenic nature of this process. In contrast to the uptake of pathogens or FcR-mediated phagocytosis, the engulfment of apoptotic cells does not induce inflammatory cytokine production. Currently is widely accepted that phagocytosis of apoptotic cells by LPS activated macrophages induce secretion of the anti-inflammatory and immunoregulatory cytokine IL-10 and decreases the secretion of inflammatory cytokines like TNF-α, IL-1β and IL-12 [104]. Additionally, macrophages that have engulfed apoptotic cells *in vitro* secrete TGF-β, which has been considered as a central player in anti-inflammatory responses [105]. Interestingly, liposomes exposing PS restored the TGF-β1 production when cells not expressing PS fail to induce it previously, suggesting an important role for this anionic phospholipid as immune suppressor [106]. Taken together, PS seems to be the major “eat me” signal and immune suppressor in the clearance process of apoptotic cells. 

Since millions of cells die constantly in multicellular organisms and since there is a robust system for their rapid recognition and removal, the “silent clearance” of the apoptotic cells is thought to participate in some conditions associated with an impaired cell-mediated immunity and increased apoptosis. Cancer, exposure to radiation, and some parasite and viral infections are considered to take advantage of this ubiquitous mechanism. Apoptotic promastigotes from *Leishmania* parasites induce release of TGF-β by neutrophils, suggesting that the presence of apoptotic parasites provides survival advantage for the viable parasites fostering disease development [107]**.**

### 4.4. Consequences of a failure in the clearance of apoptotic cells

Apoptotic cell removal is mainly accomplished by a widespread phagocytic system comprising macrophages, dendritic cells, Kupffer cells, microglia, and alveolar macrophages. If apoptotic cells are not efficiently removed, cellular membranes may disrupt releasing intracellular danger signals that may exert immune stimulatory effects [108]. Deficiencies in the elimination of dying cells may thus promote the development of chronic autoimmune diseases as described for systemic lupus erythematosus (SLE). We demonstrated that macrophages from patients with SLE are impaired in the phagocytosis of autologous apoptotic material *in vitro* [109]. Furthermore, in lymph node sections of some patients with SLE, the number of tingible body macrophages containing ingested apoptotic material was significantly lower. Furthermore, the macrophages lacked the typical morphology and were smaller than those found in lymph nodes of non-SLE individuals. In these patients, apoptotic nuclear remnants were observed to be associated with follicular dendritic cells in the germinal centres [110], thus putatively providing survival signals for B-cells that had accidentally developed autoreactivity during the random process of somatic mutation. The development of an antigen driven immune response against nuclear and apoptosis related autoantigens is the first direct consequence of the accumulation of apoptotic remnants due to an impaired clearance [111]. This observation is the most direct evidence about the etiology of autoimmunity in SLE. 

The second consequence of an impaired clearance implies anti-nuclear autoantibodies encountering nucleic acid-containing apoptotic remnants either in circulation or deposited in tissues to form immune complexes. Such immune complexes are then susceptible to be cleared by blood-borne phagocytes, macrophages, and DC through Fcγ-receptor-mediated phagocytosis. The latter is then associated with the secretion of high amounts of inflammatory cytokines [112]. The final outcome is multiple organ damage leading to the establishment of chronic inflammation.

## 5. Annexin A5: A Natural Ligand of PS

A natural occurring ligand for PS is Annexin A5 (AnxA5), a 35.7 kDa protein that belongs to a huge family of evolutionary related annexin proteins detected in most eukaryotic *phyla* [113]. Each annexin is constituted of two different regions, the unique N-terminal domain, also called the "tail", and the C-terminal domain, named "core". The core domain of annexins consists of four similar repeats approximately 70 amino acids long with the exception of Annexin A6, which has eight repeats. In general, the core domain is responsible for the binding of Ca^++^ and phospholipids [see [114] for an extensive review]. Annexins are believed to exert various functions in inflammation, membrane trafficking, opsonisation and phagocytosis, inhibition of coagulation, transmembrane channel activity, transduction of mitogenic signals, cell-matrix interactions, and they may also serve as stress proteins [115]. The tertiary structure of the soluble form of Annexin A5 has been solved with X-ray crystallography [116,117,118]. The core of AnxA5 consists of four domains arranged in a cyclic array. AnxA5 binds reversibly with high specificity to PS-expressing membranes in a Ca^++^-dependent manner and the use of fluorescent labeled AnxA5 is currently the gold standard method for detection of apoptosis by flow cytometry [119,120]. AnxA5 is present in both intracellular and extracellular milieu. The concentration in the circulation is approximately 1.5 nM. The ionized calcium concentration in the circulation, which is approximately 1 mM, favors a rapid binding of AnxA5 to cell surface-expressed PS. Binding of AnxA5 to membranes is not only determined by the presence of PS, but also by the presence of other phospholipids such as PE [121]. As mentioned above, there are also viable cells exposing increased amounts of PS. In order to understand this controversial feature of PS exposure, we analyzed the binding of AnxA5 to viable and dying monocytes. We found that AnxA5 interaction with apoptotic and necrotic monocytes proceeds in a co-operative manner whereas the binding to viable monocytes not. This suggests that cell membranes of dying cells have a higher lateral mobility of PS and that AnxA5 needs a critical density or clustering of PS molecules [7].

AnxA5 has two major physiological roles *in vivo*, namely the anticoagulant activity preventing thrombotic processes [122,123], and as modulator of the immune response by inhibiting the phagocytosis during the clearance of apoptotic and necrotic cells [124]. In the first case, it has been proposed that AnxA5 inhibits the formation of the prothrombinase complex and consequently of thrombin by forming a two-dimensional lattice on the PS-expressing surface [125,126,127,128]. In the second case, AnxA5 has been shown to be capable to disturb apoptotic cell clearance by blocking PS. Furthermore, it has been shown that after PS binding, AnxA5 crystallizes as an extended two-dimensional network resulting in the internalization of the PS-expressing membrane patches. This additionally reduces the availability of PS on apoptotic cells for phagocytes [129].

### 5.1. Annexin A5 in cancer therapy

To further characterize clearance disruption and immune modulation induced by AnxA5, we used a knockout mouse (KO) model which allows addressing *in vivo* functions of AnxA5 [130]. Wild type (WT) mice immunised with allogeneic necrotic (mechanically stressed) cells showed a strong delayed type hypersensitivity reaction. In contrast, AnxA5-deficient animals displayed a strongly decreased reactivity against dead allogeneic cells [131]. Additionally, an increased secretion of the anti-inflammatory cytokine IL-10 of isolated macrophages of AnxA5 KO mice was observed. Conversely, in WT mice, where endogenous AnxA5 is present, activated macrophages secreted higher amounts of TNF-α and IL-1β while the amount of TGF-β was lower. We also observed that tumour size of allogeneic CT26 colorectal tumor cells regressed faster compared to AnxA5 KO mice [132]. These results were also observed using a syngeneic mouse model. The addition of AnxA5 to irradiated apoptotic tumour cells, used as tumour vaccine, increased the percentage of tumour-free mice in syngeneic tumour models and AnxA5 alone led to a retardation of syngeneic tumour growth [133].

Normally, apoptotic cells are poorly immunogenic. This property can be used by tumour cells to escape from the immune system creating a local immunosuppressive milieu defined by IL-10, TGF-β, soluble FAS and FAS-ligand, as well as soluble PS [15]. Blocking the clearance of apoptotic tumour cells by exogenous AnxA5 may open a new strategy for developing tumour vaccines. We found that the disruption of the c1earance of apoptotic tumour cells by AnxA5 may trigger a pro-inflammatory response contributing to a specific immune reaction against tumor cells. Incubation of apoptotic cells with AnxA5 prior to immunisation significantly increased the immunogenicity of the cells undergoing apoptosis [134]. Interestingly, AnxA5 decreased apoptotic cell uptake by peritoneal macrophages and increased their uptake by dendritic cells [133,135].

These data suggest that AnxA5 influences the phagocytosis of dying cells and modulates the immunological response against both allogeneic and syngeneic cells. This mechanism may be employed for future cancer therapies aiming to induce a specific immune reaction, a reduced tumour-load, and a long-lasting anti-tumour immunity by combining standard therapies with the application of exogenous AnxA5.

### 5.2. Annexin A5 in infections

**Figure 4:**
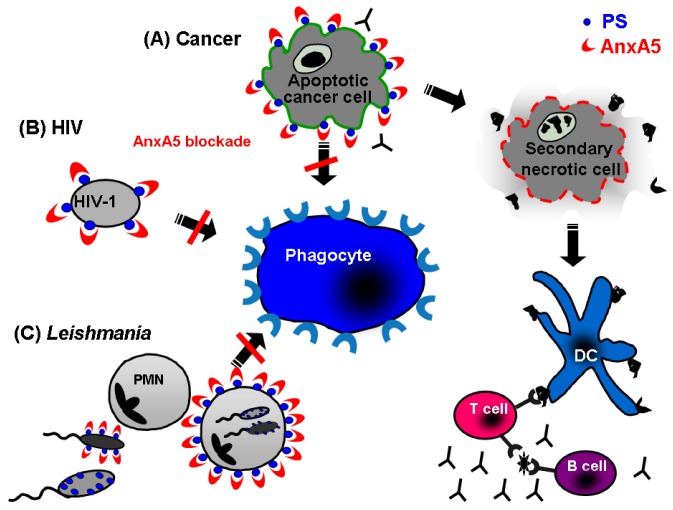
Annexin A5 as modulator of immune responses.Apoptotic cells expose PS leading to an efficient clearance by macrophages. The interaction of apoptotic cells with macrophages can be blocked by AnxA5, a specific ligand for PS. Therefore, AnxA5-based therapy strategies result in the accumulation of secondary necrotic cells. Under those circumstances, dendritic cells get the chance to take up dead-cell derived antigens, and are co-stimulated by danger signals released from secondary necrotic cells. The presented antigens lead to activation of T cells, which provide survival signals for B cells leading to a specific immune reaction against apoptotic cell-derived antigens. This strategy is useful to improve immune reactions against cancer cells (A) as well infectious agents like PS exposing virus (B) and Leishmania parasites (C), which use PS exposure as a tool to improve their survival by fooling the immune system.

It has been shown that many viruses can induce both apoptosis and PS exposure in the infected cells. PS can also be found in the outer membrane of enveloped retrovirus [136]. We studied the influence of AnxA5 on chronic macrophage infection with HIV-1, known to expose PS on its surface. We found that infectivity in human macrophages of HIV-1 was significantly reduced in the presence of AnxA5 [131]. Zandbergen and colleagues explored the role of PS during *Leishmania* disease and discovered that virulent inoculums of *Leishmania* promastigotes contained a high ratio of PS exposing apoptotic parasites. However, after apoptotic parasites depletion from the virulent inoculum, *Leishmania* did not survive in phagocytes *in vitro* losing their disease inductor capacity in vivo [107]. In summary, AnxA5-based therapy strategies may be also useful to improve immune reactions against various infectious agents which use the PS exposure as a tool to improve their survival by fooling the immune system as well as against apoptotic cancer cells (Figure 4).

## 6. Final Perspectives

**Figure 5:**
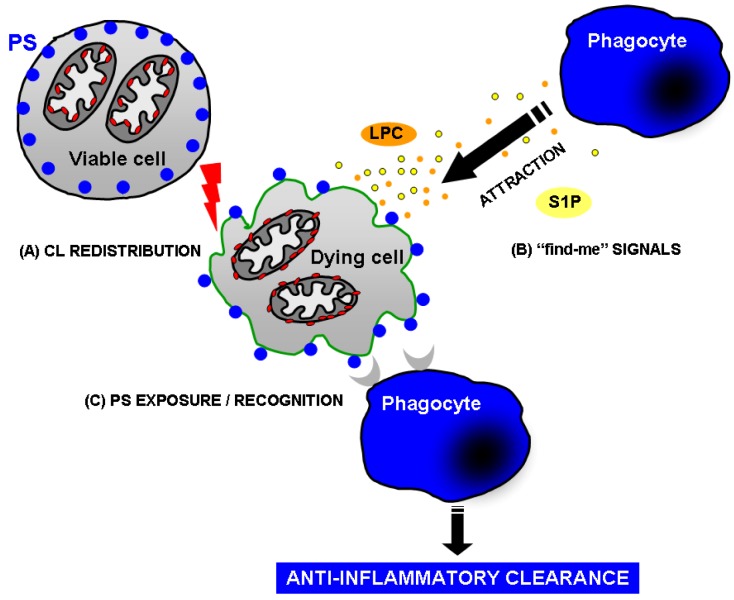
Role of cardiolipin, phosphatidylserine, lysophosphatidyl-choline, and sphingosine-1-phosphate during apoptosis and apoptotic cell clearance.The involvement of phospholipids during apoptosis is depicted: (A) Cardiolipin (CL) translocation from IMM to OMM, finally resulting in the cytochrome c release, loss of the mitochondrial potential membrane, and gradually stopping of ATP synthesis. (B) Chemoattraction of phagocytes by a gradient consisting of lysophosphatidylcholine (LPC) and sphingosine-1-phosphate (S1P). (C) PS translocation to the external cellular membrane. PS is the main “eat me” signal for apoptotic cells.

The distinct role of phospholipids during apoptosis is complex and still under intensive investigation. However, one can summarize three main events (Figure 5): (1) *CL translocation from IMM* to OMM: exposure of CL to the OMM causes a plethora of events finally resulting in the release of cytochrome c, loss of the mitochondrial membrane potential, and consequently block of ATP synthesis. This process is associated with the so called “intrinsic apoptotic pathway” and can be induce by both internal (e.g., DNA damage) and external stimuli (cell-death activation receptors). (2) *Attraction of phagocytes:* this is mediated by several factors, like LPC and S1P, which have been identified as important players in the establishment of chemoattraction gradients necessary for the swift finding and engulfment of apoptotic cells. (3) *PS translocation to the external cellular membrane:* PS is considered as the main “eat me” signal for phagocytes of apoptotic cells.
